# The crime scene reconstruction of the shrapnel effect on human body by two hand grenades detonated in a room: a case approach

**DOI:** 10.1186/s41935-017-0011-0

**Published:** 2017-07-18

**Authors:** Ercan Seyhan, Salih Cengiz

**Affiliations:** 1 Jandarma ve Sahil Güvenlik Akademisi, Beytepe-ANKARA, Turkey; 2 Istanbul University Forensic Sciences Institute, Istanbul, Turkey

**Keywords:** Fragmentation, Blast, Detonation parameters, Peak pressure

## Abstract

**Background:**

The case relates to a bookstore owner claiming that two DM-41 hand grenades were exploded simultaneously in his store. There were three males together at the store when the explosion occurred. One was the owner who claimed that he escaped after the explosion without any harm; the other was at the corner lying down to prevent his body from the explosion effect. He survived with very minor, almost no effects.

**Case presentation:**

According to the hospital report, it was stated that "cuts on the right femur with sizes of 0.5x2 and 0.5x1 cm and one cut of 0.5x2,0 cm on the left food which are curable with simple medical intervention; generalized skin erosions on body with the sizes between 0,5 to 1,0 cm"; the third male was standing and killed. He was next to the lying down male. At the autopsy report it was stated that the he was killed due to the shrapnel/fragmentation effect, breaks on humerus, radius, femur and cranium; cerebral and internal hemorrhage.

The males witnessed at the court that they had survived with no vital damage on their bodies, they had seen the perpetrators and heard them talking. With the fact that the deceased male was intensively affected with the fragmentation/shrapnel due to the autopsy report, it was the court’s wonder if it is possible for the survived men to have no or very minor nonfatal fragmentation effect on their bodies even being in the same room with the deceased.

**Conclusion:**

It was mainly aimed to test the fragmentation effect of 2 DM-41 defence hand grenades when detonated in a closed environment (an empty room with the approximately same size of the related case). The test room was empty with no secondary fragmentation sources as window glasses etc. 3 male mannequins were used as test materials. With the post blast reconstruction of the crime scene, it was aimed to determine if the test results and the autopsy report are very coherent and the persons having the direct blast effect would be expected having maximum exposure to the fragmentation.

## Background

The degree of the explosion effect on environment and body is evaluated by the explosive type used and detonation parameters. The death and woundings took place due to the direct blast or blast reflection and the primary and secondary fragmentation effects. In addition to the fragmentation/shrapnel effect, broken windows and concerete etc. are the causes for severely wounding and deaths too. The severity of blast effect on human body depends mainly on the blast parameters and the duration of blast exposed which means the bigger the effect the worse the damage is. The other main factor is the distance to the detonation center. The closer to the explosion center is the cause for being exposed to the peak pressure longer which means that the bigger damage on body exists (Glover, 2002).

The blast effect of post explosion causes severe woundings and deaths at very vicinity of explosion centers. It is the scientific fact that the post blast has effects on the shrapnel and other solid formations to shatter and fly around. So, the sensitivity to the blast and fragmentation depends on the distance to the explosion center and the fragmentation (Glasstone & Dolan, 1977; Sartor, 1983; Manual, 1990).

The lungs are particularly susceptible to damage due to the extensive air/lung tissue interfaces (Yelverton, 1997). It is the most common fatal injury caused by the primary blast injury among the initial survivors of the explosion. An overpressure of about 40 psi will cause lung injuries. The most common lung injury associated with a blast wave is a pulmonary contusion. It appears to be more common on *the side closest to the explosion, but this may be influenced by the geometry of the surrounding area and reflected energy* (Maynard et al., 1997; Sharpnack et al., 1991; Mayorga, 1997). The threshold value for lung damage is *12 psi* and fatal effect is 40 psi (Glover, 2002)*.*

At a pressure of about 35 kilopascals (5 psi), the human eardrum may rupture. With an overpressure of 100 kPa (14 PSI) almost all eardrums will be ruptured. The treshold blast value for eardrum burst is *5 psi;* with extremely high pressures, the drum may be destroyed and the ossicles dislocated or fractured (Cohen et al., 2002).

Fragmentation effect can surely be effective both explosion vicitiny and long distances. The human body tolerance is very limited to the fragmentation/shrapnel effect. The scientific studies stated that the efficient minimum velocity value for a particle to penetrate the human skin is determined as 30 m/s (100 ft./s) depending on the area of the hit on the body and the particle weight (Richmond et al., 1968)*.* The other main reasons for being wounded or death are the structure collapses, broken windows and debris causing fragmentation effect with enough blast and velocity. The blast value for the windows break is *≤0,5 psi* (Braise & Simpson, 1968 *)*.

The victims of primary blast injury almost always have other types of injury, such as penetrating wounds from flying debris or blunt trauma from impact on immovable objects (Cernak et al., 1999). Explosions near or within hard solid surfaces become magnified 2–9 times as the shock wave is reflected (Rice & Heck, 2000). In fact, victims located between the blast and a building generally suffers 2–3 times the degree of injury that an individual in an open environment would receive (Boffard & MacFarlane, 1993). A blast wave that would cause only modest injury in the open can be lethal if the victim is in a confined area or near a reflecting surface such as a solid wall or a building (Elsayed, 1997). Secondary blast injury is much more common than primary blast injuries. Indeed, secondary blast injury is the most common cause of death in blast victims. The penetrating injuries occur most often in the exposed areas such as the head, neck, and extremities. Thoracic and intraabdominal injuries may occur when fragments penetrate (Almogy et al., 2002).

## Case presentation

The case relates to a bookstore owner claiming that two DM-41 hand grenades were exploded simultaneously in his store. He pointed out that he had seen two persons were running away after throwing the grenades into the store. After seeing the hand grenades on the floor, one is in the middle and the other near the door, he fled toward the door. The explosions took place when he was just leaving the store door and running to the corridor with many windows on the both sides.

**Fig. 1:**
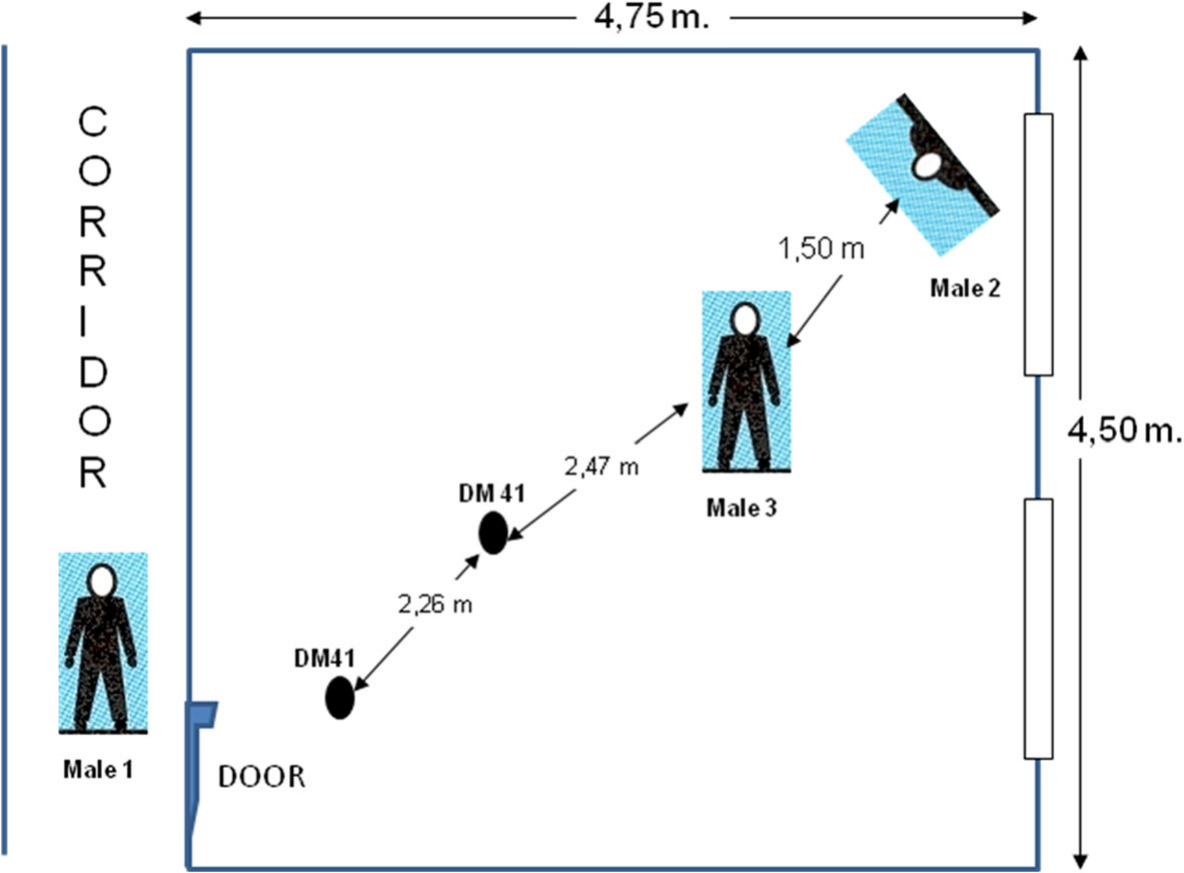
Sketch of the case with two DM-41 hand grenades were detonated

There were three males together at the store when the explosion occurred. One was the owner *(male 1)* who claimed that he escaped after the explosion without any harm; the other *(male 2)* was at the corner lying down to prevent his body from the explosion effect. He survived with very minor, almost no effects. According to the hospital report, it was stated that “cuts on the right femur with sizes of 0.5x2 and 0.5x1 cm and one cut of 0.5x2,0 cm on the left food which are curable with simple medical intervention; generalized skin erosions on body with the sizes between 0,5 to 1,0 cm”; the third male *(male 3)* was standing and killed. He was next to the lying down male (Fig. 1).

**Fig. 2:**
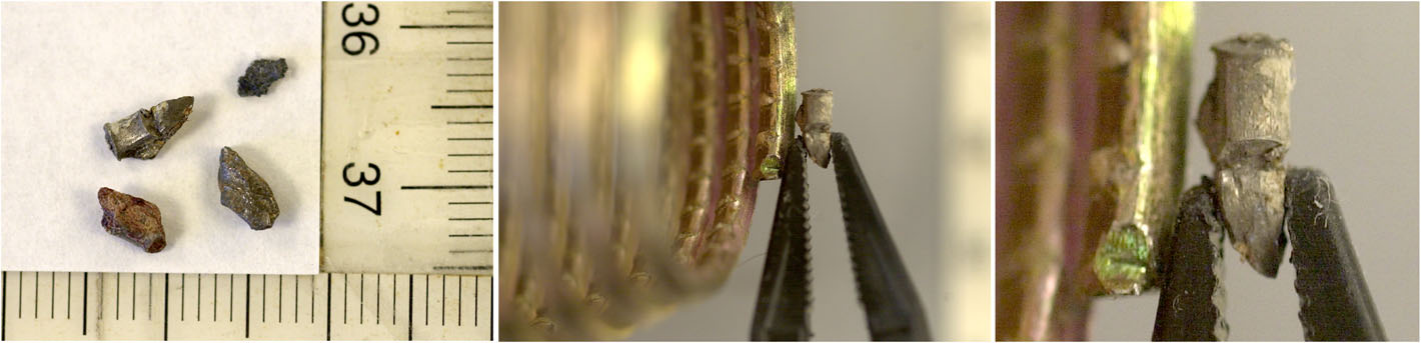
Metals taken out from the deceased body and comparison to DM-41 steel fragmentation sleeve

At the autopsy report it was stated that the *male 3* was killed because of fragmentation/shrapnel effect. The cause of death was stated as *“due to the shrapnel/fragmentation effect, breaks on humerus, radius, femur and cranium; cerebral and internal hemorrhage”.* Some metals taken out of the deceased body were sent to the forensic laboratory and determined as belonging to the DM-41 hand grenade fragmentation sleeve (Fig. 2).

*Male 1* and *male 2* witnessed at the court that they had survived with no vital damage on their bodies, they had seen the perpetrators and heard them talking. With the fact that the deceased *male 3* was intensively affected with the fragmentation/shrapnel due to the autopsy report, it was the court’s wonder if it is possible for the male 1 and male 2 to have no or very minor nonfatal fragmentation effect on their bodies even being in the same room with the deceased.

## Post mortem examinations

*According to the* post mortem *examination results (Diyarbakir Office of the Chief Prosecutor, November 10th, 2005 and investigation number 2005/2855) are as follows;*

### External examination

*1. The shrapnel wounds were determined; (a) Total of 20 with different sizes at head, neck, arms and hands, (b) Many with the sizes between 0,1 to 1,5 cm at right leg, hip, femoral and feet, (c) Many with the sizes between 0,1 to 1,0 cm at left leg, hip, femoral and feet.*

*2. Multi-piece fracture and deformities at upper right orbita, upper left first tooth, 1/3 upper right humerus, bottom right femur and bottom right radius,*

*3. The right eye emptied from bulbus oculi.*

### Classical autopsy

*1. It was distinguished; (a) ecchymosis under head skin, (b) break at outher lamina, (c) subrocnoidal bleeding at both hemisphere (d) rough edged bone defect at inner lamina,*

*2. Many subpleural bleeding with sizes between 0,3 to 2,0 cm on lung and congested cuts,*

*3. Generalized subcutan bleeding on the left neck soft tissue,*

*4. In obdomen; free blood at hepatic lobe and full cut wound at right bottom edge liver,*

*5. At extremities inspection; (a) four demormed metal pieces were taken out from right deltoid and right femur, (b) breaks on humerus, radius, femur and cranium, (c) cerebral and inner hemorrhage due to internal organ woundings, (d) fatal shrapnel wounds at head frontal hairy part and right subcostal area.*

## Discussion

The main fragmentation effect of the explosion, *primary fragmentation*, comes from the shrapnels. The particles propelled from the explosion seat and the environment is the *secondary fragmentation*. The blast mostly makes the buildings collaped and windows broke. The velocity of the blast travel in the air takes the free particles on and travels far away with tremendous speed and energy creating pressure of thousands of pascals which causes fatal wounds on the body*.* As the distance from the blast epicenter increases, the effect of blast reduces and the effect of fragments and debris propelled by the explosive becomes more important. Conventional military explosives may create multiple fragments with supersonic initial velocities (Glover, 2002).

If the explosion takes place in a closed environment with glasses, then the broken windows travels in the air with tremendous velocities and energy. This is one of the main reasons for killings and woundings. The pressure needed for a window breakage is *≤0,5 psi* Maynard et al., 1997.

These flying projectiles can produce both penetrating and blunt trauma, depending on the size of the projectile and the speed at which they travel. With these velocities, the victim does not have to be in close proximity to the explosion. Individuals far from the scene of an explosion can be struck and injured by this debris (Wightman & Gladish, 2001). The human tolerance treshold for these effects are limited. *At the literature was stated that the velocity nedded for a particle to penetrate the human skin is approximately 30 m/s (100 ft./sn.)* Seyhan, 2015.

Fragmentation type hand granades were designed to kill and give damage to people to a defined distance. This is the fact that it can cause fatal woundings and killings when detonated near living beings. *The threshold value for ear drum rapture is 5 psi; lung damage is 12 psi and fatal wounding is 40 psi* Glover, 2002.

The death and woundings took place due to the direct blast or blast reflection and the primary and secondary fragmentation effect. In addition to the fragmentation/shrapnel effect, broken windows and concerete etc. are the causes for severely wounding and deaths too. The shrapnels from the grenade body and the secondary fragments from environment travel at air by the effect of detonation blast and give fatal damages. Glass causes many of the secondary blast injuries (up to 50% of all blast injuries). Victims that are peppered with glass are often difficult to distinguish from victims that are peppered with glass and have penetrating injuries (White et al., 2008; Wong et al., 1997).

Tertiary blast injuries are caused when the victim’s body is propelled into another object by the blast winds. Tertiary effects result from the bulk flow of gas away from the explosion. Blast winds can generate a body acceleration of over 15 g’s. They most often occur when the victim is quite close to the explosion (Candole, 1967; Stuhmiller et al., 1991). This displacement of the victim can take place relatively far from the point of detonation if the victim is unfortunately positioned in the path where gases must take to vent from a structure, such as a doorway, window, or hatch.

With this respect, the possible effects expected for DM-41 defence hand grenade in a closed environment like a room can be assessed as follows;

### Possible effects inside the room

**Fig. 3:**
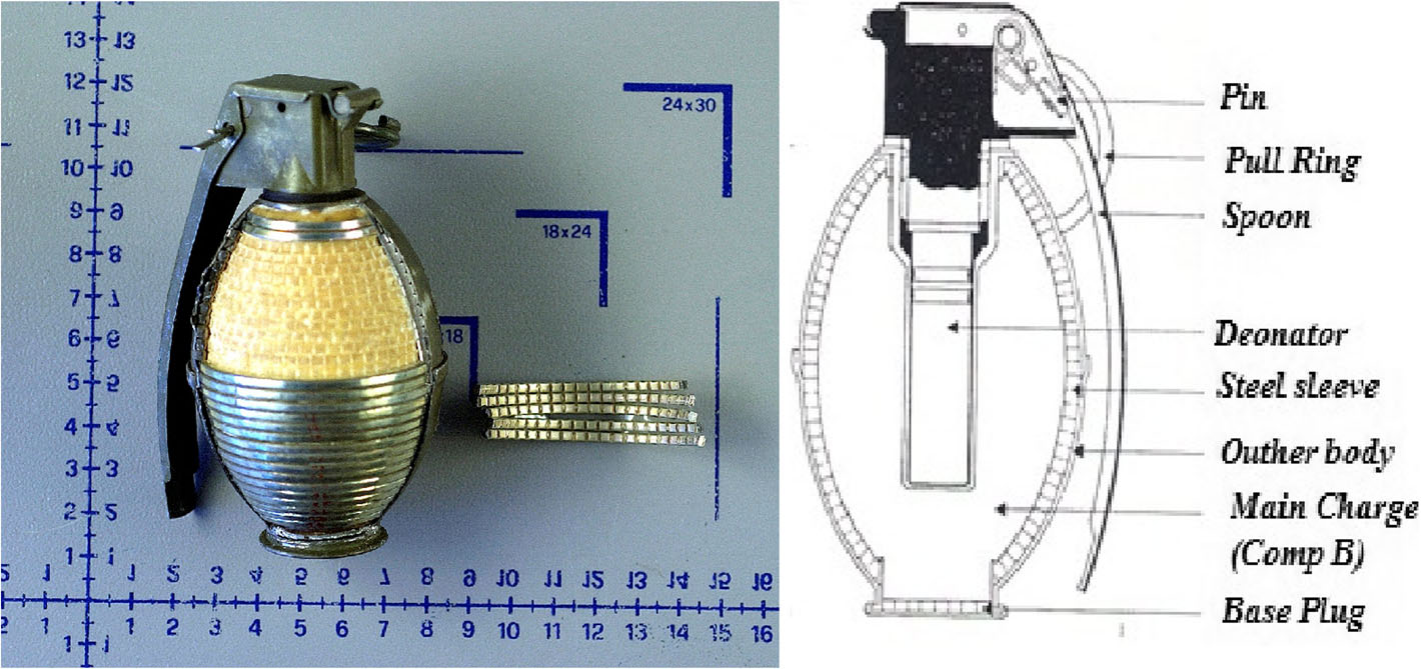
DM-41 hand grenade steel sleeve and DM-41 hand grenade cutaway

(a) It was positively defined at the forensic laboratory that two *DM-41 model fragmentation type* hand grenades were detonated in the incident site. The main charge used in these grenades is 165 g of *Composition B (60% RDX + 40% TNT). Composition B (60% RDX + 40% TNT) detonates* with 7900 m/s detonation velocity and applying 268 kPa (38,870,137,116 psi) peak pressure with a fatal radius of 20 m. The fragmentation effect is provided with a grooved steel sleeve (Bailey & Murray, 1989). The steel sleeve has 31 wrap with 1000–1010 grooves on it. The base plug also has 33–36 round shape steel fragmentation on it (Fig. 3).

(b) Two DM-41 hand granades originally designed for providing fragmentation effect have total of 2000–2100 grooved steel fragments (including the base plug). The initial detonation velocity is 7900 m/s and the blast value is 268 kPa (38,870,137,116 psi).

**Fig. 4:**
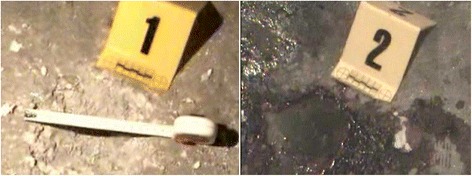
The explosion seats belong to detonated two DM-41 hand grenades

This means that two handgrenades detonated inside a room with a peak pressure of 268 kPa (268,000 Pa pressures on 1 m^2^) in very less than a second and the blast wave started to travel inside room with an initial velocity of 7900 m/s (Fig. 4).

**Fig. 5:**
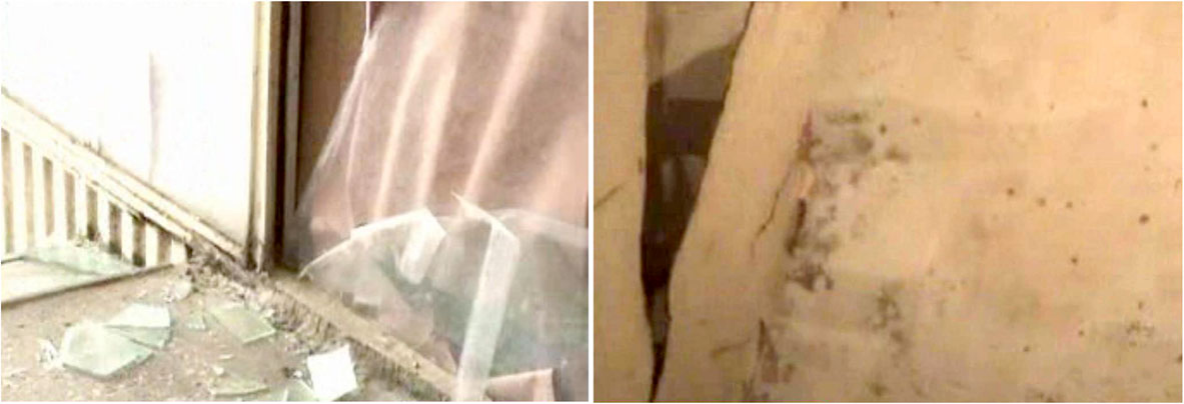
Broken windows and wall damages

(c) The shock front with supersonic velocity will move inside the room and hit the walls and be reflected. It will breake the windows and gives damages to the walls (Fig. 5).

**Fig. 6:**
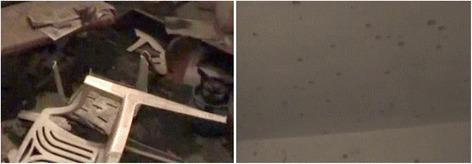
Dragging the free particles away and causing shrapnel effect on the walls and ceiling

The particles and the broken windows will be expected to propel around dragging the free particles away all causing shrapnel effect on its way (Fig. 6).

### Possible effects on living beings

**Fig. 7:**
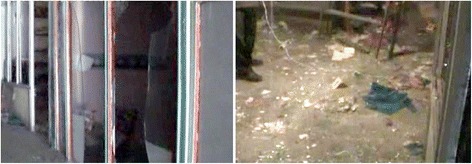
Shock wave traveling on the hall and room

 (a) Two handgrenades detonated inside a room with a peak pressure of 268 kPa (268,000 Pa pressures on 1 m^2^) in very less than a second and the blast wave started to travel inside room with an initial velocity of 7900 m/s.

(b) 2000–2100 grooved steel fragments with the dragged metals, stones and broken windows will travel along the shock wave with an initial velocity of at least 7900 m/s and peak pressure of 268 kPa *(~38,87 psi)* (Fig. 7)*.*

As the fatal wounding threshold value is around 40 psi and the peak pressure for DM-41 hand grenade is 268 kPa (~38,87 psi), it is definite that the value is between the limits of fatal wounding. So, it can be stated that a human inside this room will suffer the initial velocity and peak pressure at the highest level if not concealed behind a conceret, steel or other likewisw concelements.

(c) Propelling and severe dragging of the human body exposed to the blast effect is one of the main reasons for post blast killings and woundings. The physical injuries occur not only for the direct blast effect but also for dragging of the body*.* The walking/running person’s balance is inclined towards front. When a body running or walking exposed to a blast with supersonic speed, it is expected to lose the balance and fall. Even with the threshold value, a moving body can be expected to fall down with 268 kPa (38,87 psi) pressure and 7900 m/s.

## The post explosion reconstruction

### Material and method

**Fig. 8:**
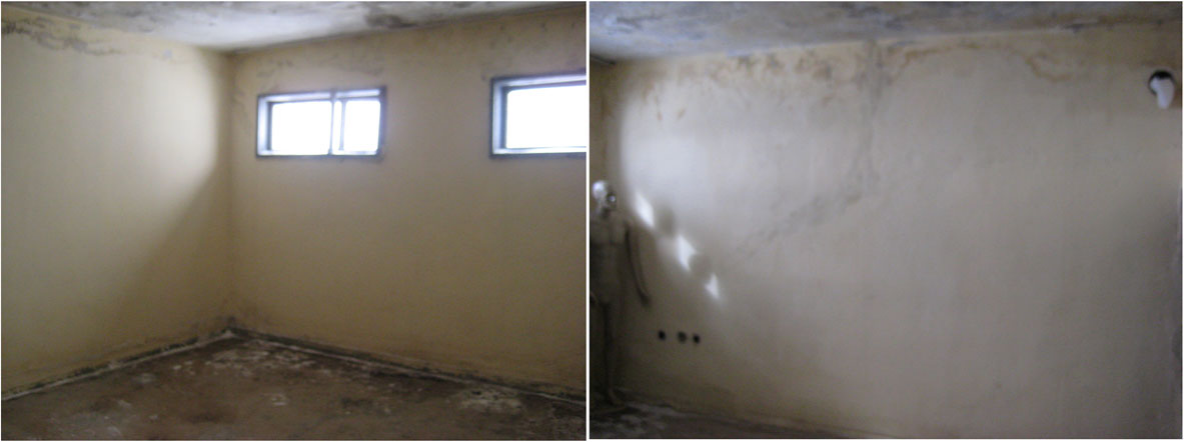
The test roomfor detonation

It was mainly aimed to test the fragmentation effect of 2 DM-41 defence hand grenades when detonated in a closed environment (an empty room with the approximately same size of the related case). The room dimensions for the test explosion was 4,5 m × 4,75 m lenghts and 3 m height (Fig. 1). The test room was empty with no secondary fragmentation sources as window glasses etc. (Fig. 8)*.*

Three male mannequins were used as test materials. Mannequins were filled with sand with an amount of normal male weight between 70 and 75 kg.

**Fig. 9:**
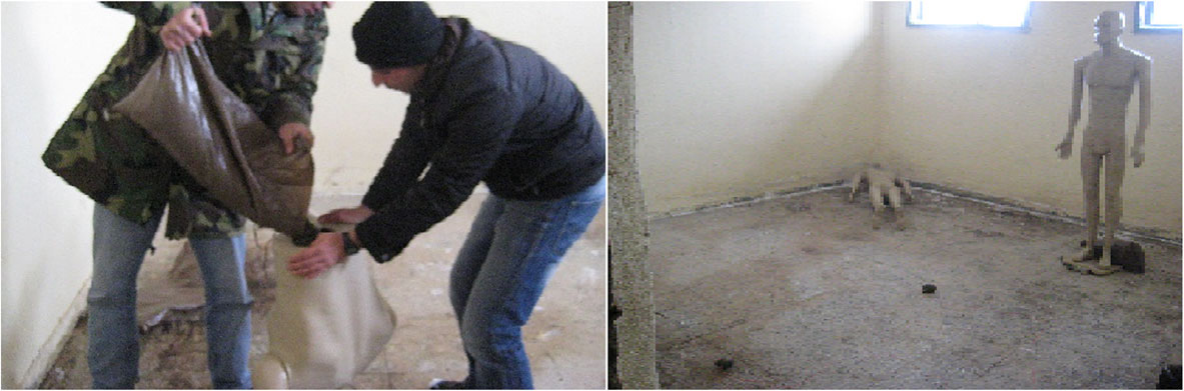
The test mannequin preparation for test detonation

The three Mannequins were positioned due to the decesead body and the other two males witnessed to the explosion case. *Mannequin-2 (male-2)* was lied face down on left opposite corne of the room due to the door. *Mannequin-3 (male 3-deceased) was positioned as standing 150 cm right and middle left of Mannequin-1. Mannequin-1 (male-1) was positioned face to the* corridor door with his left side was next to the open door (Fig. 9).

Two DM-41 hand granades were positioned in the room floor with the same distances stated in the case report (Fig. 1). In the case report the bomb seats were stated positively and there was no hesitation for the exact places where the detonation was occurred.

**Fig. 10:**
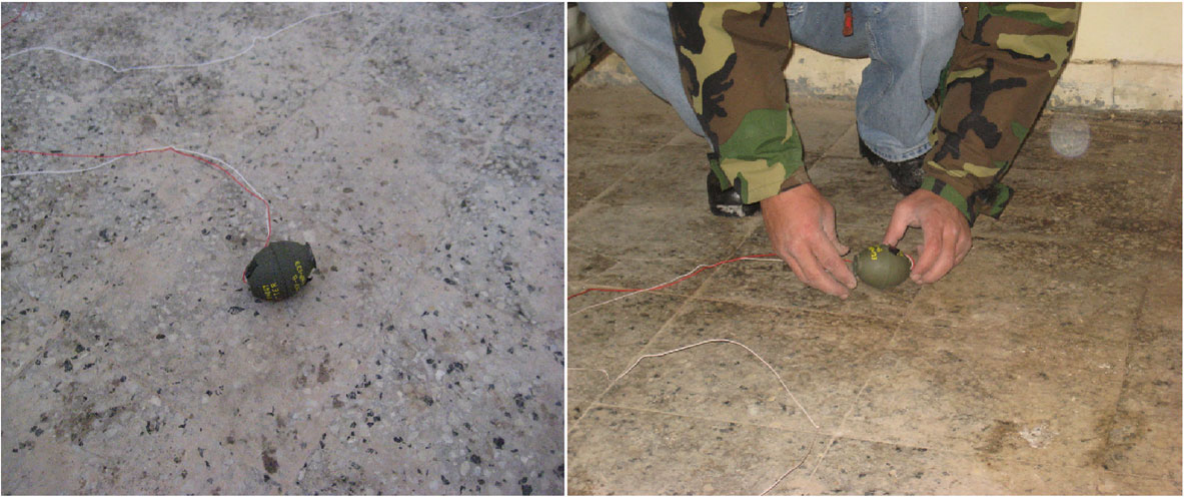
The two DM-41hand grenades primed in for test detonation

The original fuzes of the hand granades were taken out and two electrical blasting caps were primed in the fuze wells. The caps were in series. The hand granades were detonated with a positive control of the explosive (EOD) experts (Fig. 10).

**Fig. 11:**
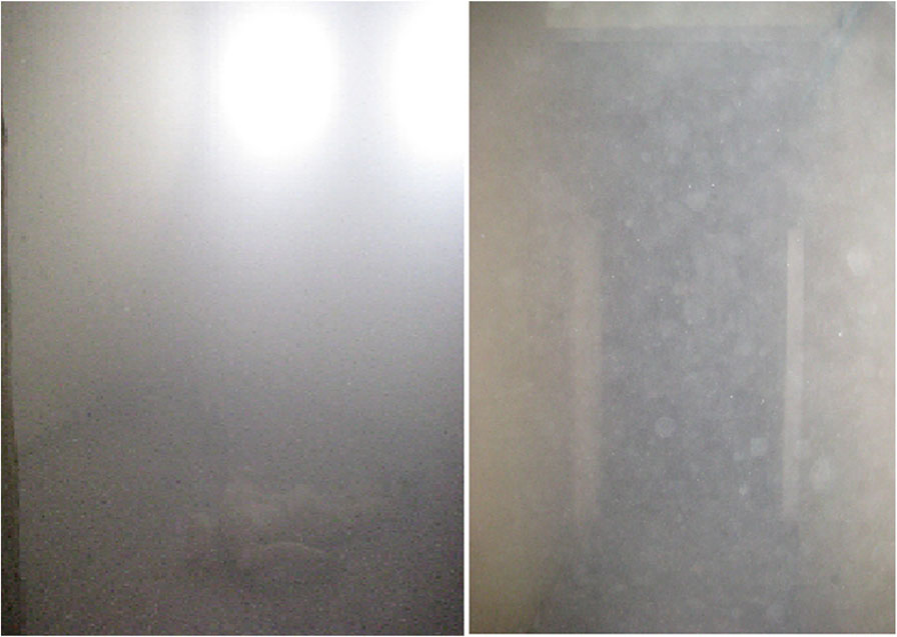
The room 10 s after test detonation

Ten secons after the detonation, we went into the room and took pictures. It was observed that the room filled with dust and the situation was choking, hard to breathe in (Fig. 11). The experts were with full protective equipment (EOD 7B Bomb Disposal Suit).

**Fig. 12:**
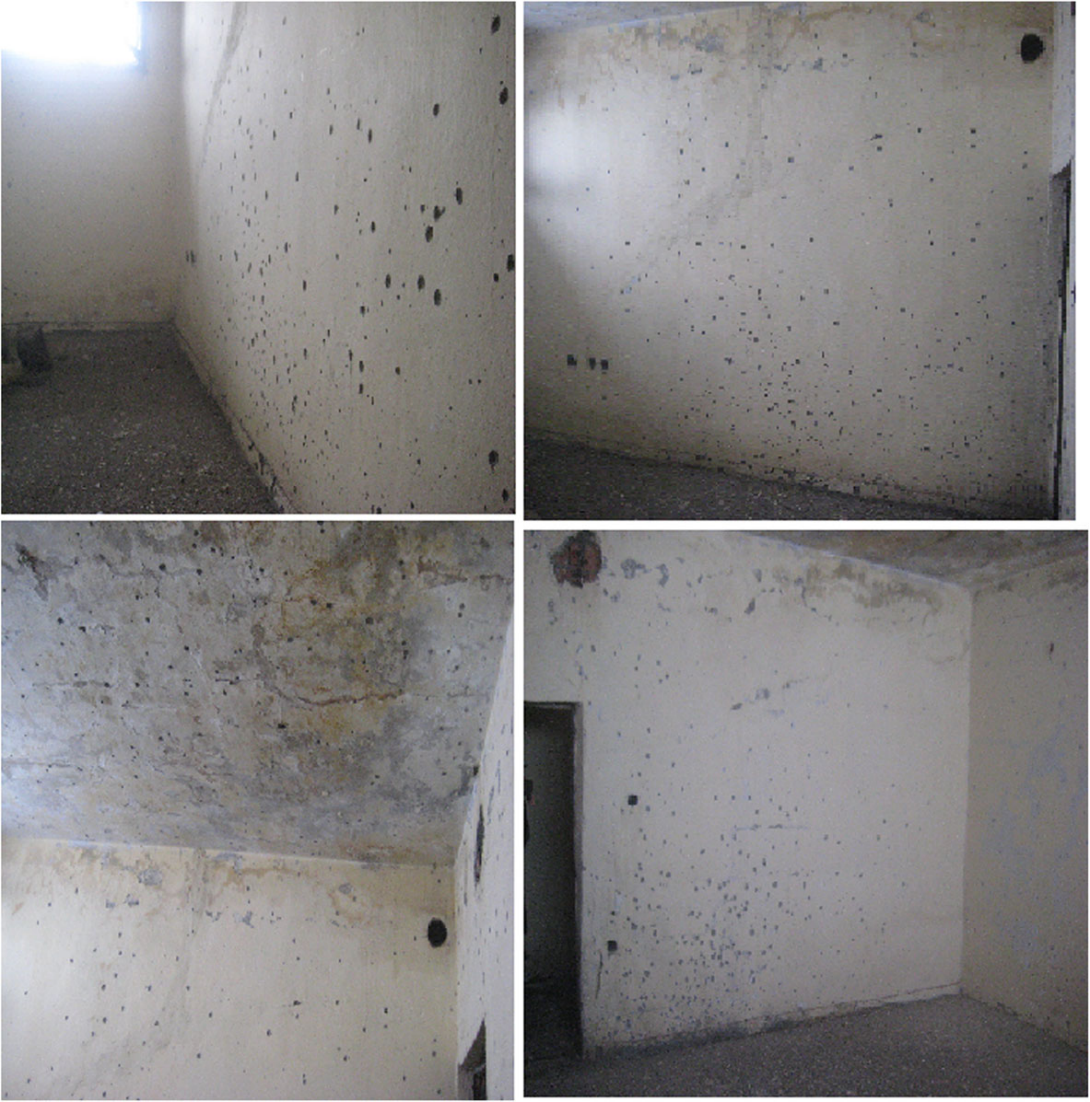
Shrapnel effects observed in test room walls and ceilings after detonation

**Fig. 13:**
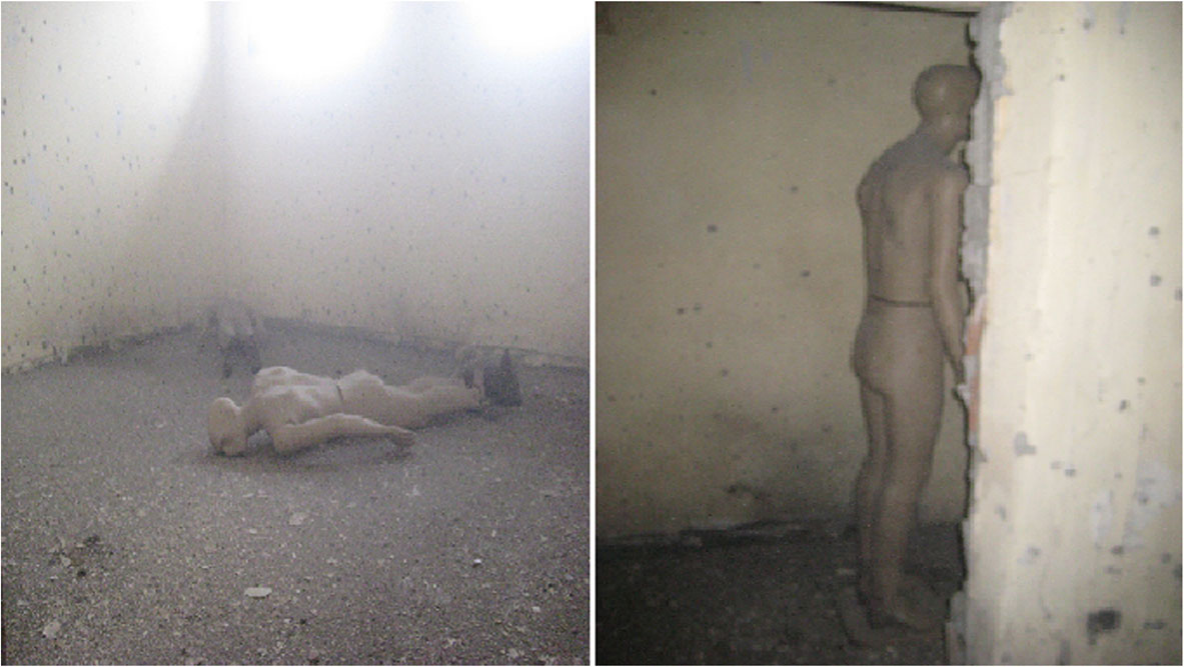
The test room after detonation

The test room was empty with no window glasses. It was carried out to test the 2 DM-41 hand granades pimary fragmentation/shrapnel effects (grooved steel sleeve) on the body (Figs. 12 and 13).

The corridor where the mannequin 3 (male-1) standing was affected just only from the shrapnel passed trough the open door. It was observed that mannequin 1 and 2 were exposed to the shrapnel effect at a maximum level.

### Findings and results

**Fig. 14:**
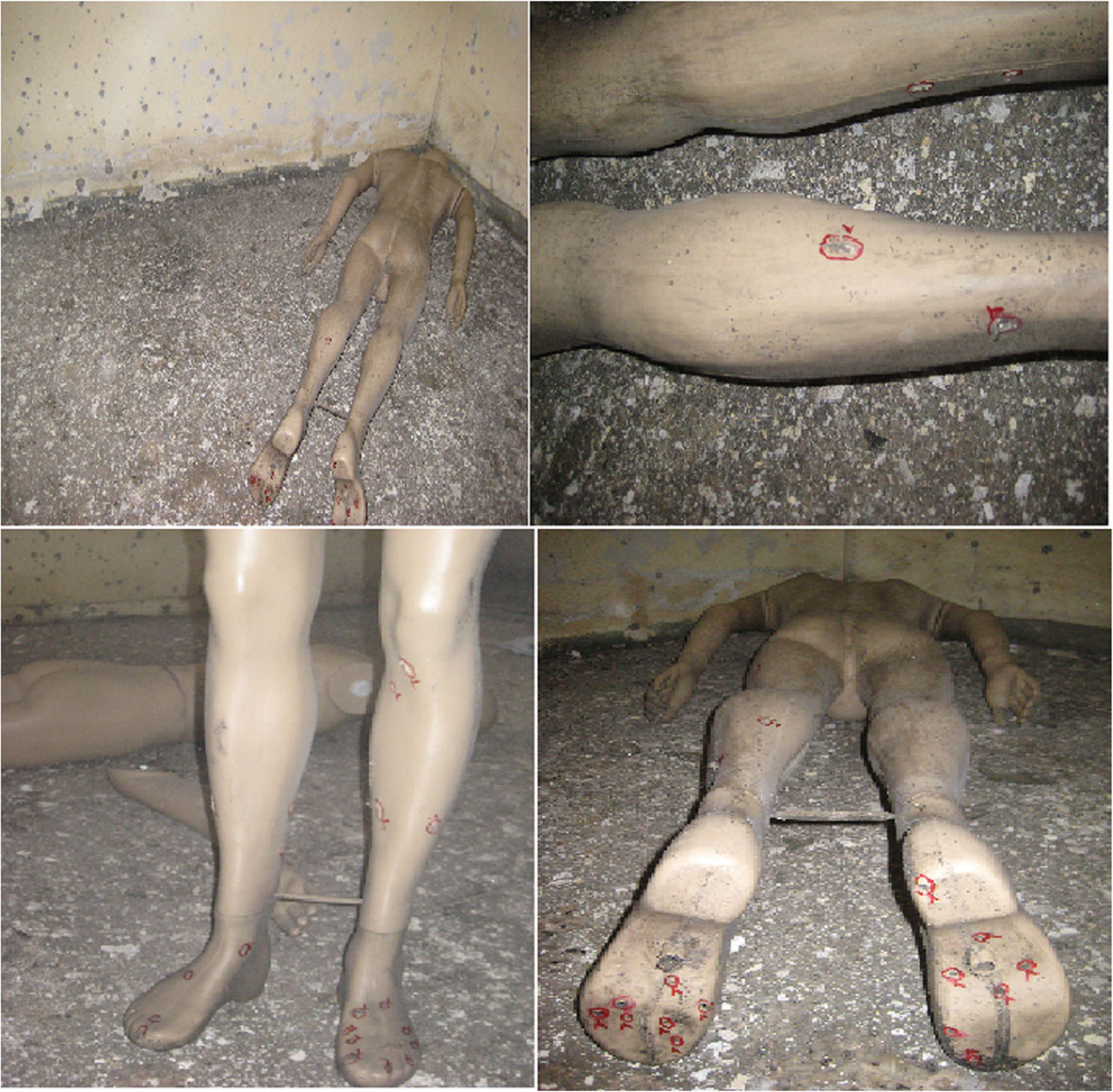
Mannequin-2 (male-2) lied *face down* on *left* opposite corner after detonation

The primary post blast fragmentation/shrapnel effects on the mannequins were stated below;

*On the Mannequin-2 (male-2) lied face down on left opposite corner of the room due to the door* (Fig. 14).

a. On the baseline of the right foot: 11 holes,

b. On the baseline of the left foot:                 16 holes,

c. On the left leg:                            9 holes,

d On the right leg:                            4 holes,

e. On the groins area:                           3 holes,

f. On the upper back:                           6 holes,

g. On the inner and outher parts of left hand:  2 holes,

h. Inner part of right hand:                           2 holes,

i. On the head:                           1 hole,

j. On the left side of chest:                            2 holes.

**Fig. 15:**
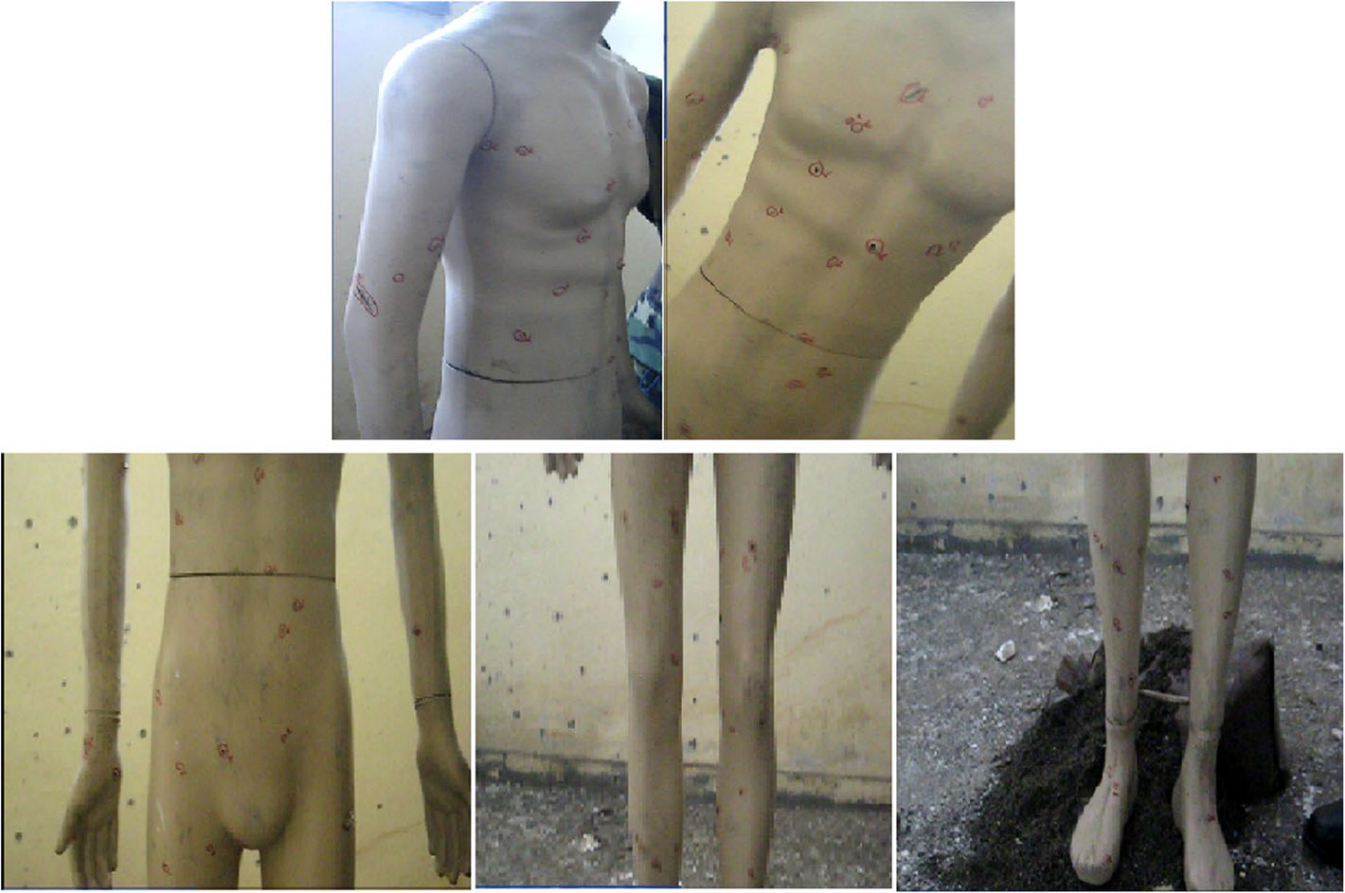
***1*** *Mannequin-3 (male 3-deceased) positioned as standing after detonation*. ***2*** *Mannequin-3 (male 3-deceased) positioned as standing after detonation*

*On the Mannequin-3 (male 3-deceased) positioned as standing* (Fig. 15-1 and 2):

a. Right side of chin:                           1 hole.

b. Left of the neck:                         1 hole.

c. Right armpit:                          1 hole.

d. Right hand and arm:                          9 holes.

e. Chest and abdomen:                           13 holes.

f. Left arm:                            4 holes.

g. Groin:                            4 holes.

h. Right leg:                           17 holes.

i. Right food:                            4 holes.

j. Left leg:                            15 holes.

k. Left food:                            12 holes.

**Fig. 16:**
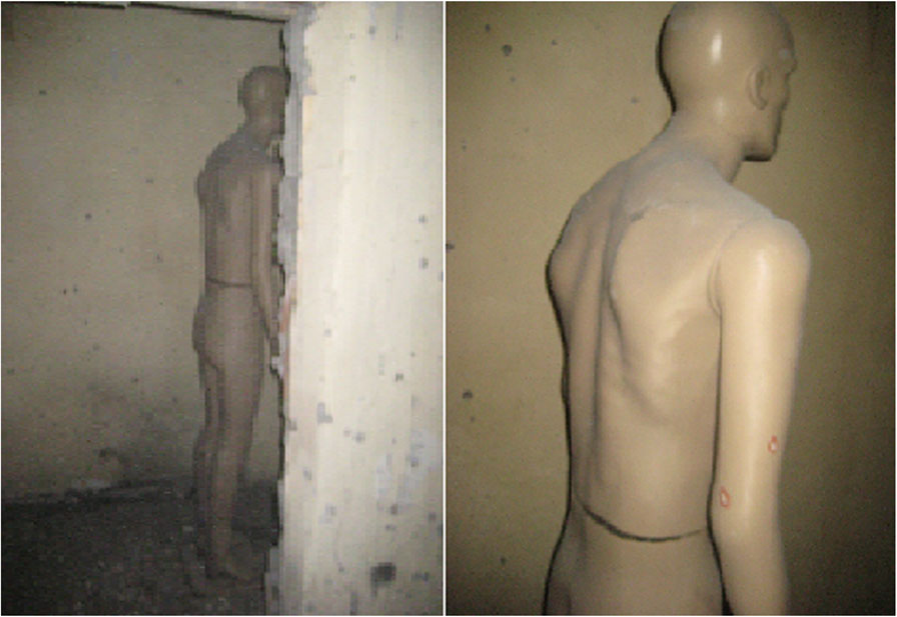
Mannequin-1 (male-1) positioned face to the corridor door after detonation

*On the Mannequin-1 (male-1) positioned faced to the corridor door* (Fig. 16)*:*

a. Right hand and arm:                            2 holes.

b. Right leg:                            1 hole.

## Conclusions

Due to the post mortem examination and test detonation results, it can be understood and stated as a post blast reconstruction of the crime scene that;

*(1). The two DM-41 hand granades were exploded in the room with aforementioned standart detonation parameters and the effects fully continued through the corridor and inside the room.*

*(2) The blast occurred at the room is very compatible with the test results and the expected standart detonation parameters for both blast and the fragmentation/shrapnel effects.*

*(3) Due to the test results and the the autopsy reports stated, it was understood that the persons very vicitnity of the explosion seats inside the room experienced the peak pressure very positively.*

*(4) The test results and the autopsy reports justified that the fragments/shrapnels from the steel sleeve of two hand granades traveled inside the room with a supersonic velocity and high pressure. The test results and the autopsy report were compatible with each other.*

*(5) The aforementioned test results justify and must be expected that the persons witnessed the explosion center would have been very well suffered the detonation as the deceased experienced. The shrapnel effect, due to the scientific findings, must be expected being compatible with the test detonation results.*

*(6) The test results and the autopsy report are very coherent that the persons inside the room and the vicinity having the direct blast effect would be expected having maximum exposure to the fragmentation if there is not a concerete or steel* etc. *barrier between, which means the other two males survived with very minor, almost no effects are not scientifically satisfactory due to the reconstruction of the case.*
